# The dysregulated expression and functional effect of CaMK2 in cancer

**DOI:** 10.1186/s12935-021-02030-7

**Published:** 2021-06-30

**Authors:** Qi He, Zhenyu Li

**Affiliations:** 1 Department of Pathology, Chongqing University Cancer Hospital, No. 181 Hanyu Road, Shapingba District, Chongqing, 400030 People’s Republic of China; 2 College of Laboratory Medicine, Chongqing Medical University, Chongqing, People’s Republic of China; 3 Department of Pathophysiology, Basic Medical College, Chongqing Medical University, Chongqing, People’s Republic of China

**Keywords:** CaMK2, Cancer, Proliferation, Metastasis, Stemness, Resistance

## Abstract

CaMK2 (calcium/calmodulin-dependent protein kinase 2), a multifunctional serine/threonine-protein kinase involved in diverse cellular processes, is vital for the transduction of the Ca^2+^ signaling cascade. Recently, research has highlighted the involvement of CaMK2 in cancer development. However, the specific effects of CaMK2 on cancer have not been fully elucidated. In this review, we summarize not only the altered expression of CaMK2 in a range of cancers, as evidenced by bioinformatics analysis, but also the significant role of CaMK2 in regulating cancer progression, such as proliferation and metastasis. In addition, we described the functional influence of CaMK2 on cancer stemness and resistance. Understanding the critical effects and mechanisms of CaMK2 in cancer would facilitate the development of a promising therapeutic strategy for cancer treatment.

## Introduction

Calcium/calmodulin-dependent kinase 2 (CaMK2), the most widely studied multifunctional serine/threonine kinase, is critical for transducing Ca^2+^ signals and is widely expressed in mammalian cells [1–3]. CaMK2 is encoded by four separate but homologous genes (α, β, γ, and δ), which produce CaMK2α, CaMK2β, CaMK2γ, or CaMK2δ, respectively [4]. The general structure of CaMK2 includes a unique N-terminal domain, followed by a catalytic domain containing an ATP-binding region, a regulatory domain that contains an auto-inhibitory region and a calmodulin-binding region, and a C-terminal association domain responsible for multimerization [5]. The canonical activation of CaMK2 requires both calcium and calmodulin. When intracellular calcium concentration rises, four calcium molecules bind calmodulin, which binds to CaMK2 and induces phosphorylation of the regulatory domain at Thr286 for the α isoform or at Thr287 for the β, γ, and δ isoforms [5, 6]. This phosphorylation induces and sustains the autonomous activation of CaMK2 in the absence of an increase in calcium [7–9]. Thus, phosphorylation of CaMK2 at T286 for the α isoform or at T287 for the β, γ, and δ isoforms has been established as a biomarker for CaMK2 activation [10, 11]. In addition to canonical activation, CaMK2 has also been activated in a calcium/calmodulin-independent manner, such as threonine 287 autophosphorylation [12], methionine 281/282 oxidation [13], serine 280 *O*-GlcNAclyation [14], and *S*-nitrosylation of cysteine 290 [15]. In addition, there are several specific inhibitors for CaMK2 activation: KN-62 and KN-93, two CaMK2 chemical inhibitors, repress CaMK2 phosphorylation by interfering binding of calcium with calmodulin, thereby suppressing CaMK2 activation [16, 17]; CaMK2N and CaMK2Nβ, two endogenous inhibitory proteins, could directly interact with CaMK2 and inhibit CaMK2 activation [18, 19].

CaMK2 has been previously implicated in diverse cellular processes [20–22], including synaptic plasticity, learning and memory, vascular smooth muscle polarization and migration, and cell proliferation. Several studies have also implicated CaMK2 in controlling the differentiation, growth, and apoptosis of cancer cells [23, 24]. Moreover, two CaMK2 chemical inhibitors, KN-62 and KN-93, were shown to induce cell cycle arrest and apoptosis, thus, decreasing the proliferation of cancer cells [25, 26]. Recently, accumulated evidence has shown that CaMK2 is strongly dysregulated in many malignant diseases and plays a pivotal role in cellular proliferation, migration, and invasion. Additionally, several recent studies have indicated the vital role of CaMK2 in the modulation of drug resistance, recurrence, and stem-like traits of cancer. These studies suggest a potentially significant role for CaMK2 in cancer progression. In this review, we highlight the dysregulated expression of CaMK2 in a series of cancers, as evidenced by bioinformatic analysis, as well as the functional influence of CaMK2 on the proliferation, metastasis, therapy-resistance, and stemness of cancer cells.

### Methods for this narrative review

The methods used for this narrative review are as follows: the databases used for the search include Web of Science and PubMed; the terms used for the search included CaMK2, CaMK2α, CaMK2β, CaMK2γ, CaMK2δ, or cancer, tumor; the types of literature retrieved from the public databases include bioinformatics articles, experimental articles, and reviews. Finally, the literature closely related to the subject of this narrative review were included. Notably, studies that met the above inclusion criteria but were withdrawn were excluded.

### The dysregulated expression of CaMK2 in cancer revealed by bioinformatic analysis

Recent studies have revealed altered expression of CaMK2 in a range of cancers with different bioinformatic analyses. Here, we discuss recently reported bioinformatic studies that show dysregulated CaMK2 expression during cancer progression (Table 1). Notably, most bioinformatics studies are only conducted based on published databases or microarray data.

**Table 1:** CaMK2 expression in numerous cancer progression is prominently changed reflected by bioinformatic analyses

Cancer types	Method of bioinformatic analysis	The altered CaMK2 expression in cancer	References
CRC	The integrated transcriptomic and proteomic datasets acquired from previous microarray- and mass spectrometry (MS)-based study	Both the transcription and protein levels of CaMK2δ are concordantly decreased during CRC progression	[27]
	The mRNA expression profile is inverstigated using GeneChip Human Transcriptome Array 2.0	CaMK2β expression is downregulated in F. nucleatum-induced CRC	[28]
	Total genome gene expression profiling is investigated using Illumina HT-12 V4.0 Expression Beadchip oligonucleotide microarray	CaMK2γ expression is significantly reduced in rectal cancer tissues compared with colon cancer tissues	[29]
GBM	The differentially expressed genes is found and validated by microarray analysis of RNA expression profiling and qRT-PCR, respectively	CaMK2β is significantly decreased in the MMLV/PDGFB mouse gliomas	[30]
	The gene expression in GBM and normal control are compared based on the data from GEPIA	The expression of CaMK2β and CaMK2γ are synchronously downregulated in GBM	[31]
	Oligonucleotide-based microarray analysis and real-time RT-PCR verification	CaMK2γ transcript level in recurrent high-grade gliomas is significantly reduced as compared to primary low-grade gliomas	[32]
	A prognostic prediction system was constructed and validated using microarray data from TCGA dataset, GEO dataset and a chinese Glioma Genome Atlas (CGGA) dataset	High CaMK2α mRNA expression may be a bad prognostic factor for GBM	[33]
HSCC	The differentially expressed genes that are closely related to the TPF chemosensitivity in priamry HSCC patients was screen using mRNA microarray analysis	The expressions of CaMK2α and CaMK2β were significantly increased in TPF-resistent patients compared with the TPF-sensitive patients	[34]
MTNBC	The differentially expressed proteins between the chemosensitivity group and chemoresistance group were identified and validated by TMT-based proteomics analysis and ELISA analysis	The protein level of CaMK2α in chemoresistance group was significantly higher than that in chemosensitivity group	[35]
NSCLC	A genome wide scan of single-nucleotide polymorphisms (SNPs) was conducted in patients with early-stage NSCLC	CaMK2δ rs10023113 was significantly associated with poor prognosis of early-stage NSCLC	[36]
Breast cancer	The expression microarray data obtained from the EBI database was screened and then identified using a combinational approach involving in silico mining followed by MSP and RT-PCR	The promoter of CaMK2β was hypermethylazed and the expression of CaMK2β was decrased in breast cancer	[37]
lingual SCC	The methylation of gene promoters in lingual SCC mouse model was determined using Methylated DNA immunoprecipitation sequencing	Significant methylation in CaMK2 promoter was confirmed in lingual SCC mouse model	[38]
EAC	Targeted RNA sequencing and PCR verification were performed in 40 paired EAC specimens and nonmalignant specimens frompatients	The USP54- CaMK2γ fusion transcripts was found in EACA specimens	[39]

*CRC* colorectal cancer, *GBM* glioblastoma multiforme, *HSCC* hypopharyngeal squamous cell carcinoma, *MTNBC* metastatic triple negative breast cancer, *NSCLC* non-small cell lung cancer, *SCC* squamous cell carcinoma, *EAC* esophageal adenocarcinoma, *TPF* docetaxel, cisplatin and 5-fluorouracil, *TMT* tandem mass tag, *EBI* European Bioinformatics Insstitute

### The decreased CaMK2 in colorectal cancer

Several original studies have shown decreased expression of different CaMK2 isoforms in CRC in the context of colorectal cancer (CRC). Hennig et al. [27] analyzed the integrated transcriptomic and proteomic datasets acquired from their previous microarray- and mass spectrometry (MS)-based studies, and found that CaMK2δ exhibited concordantly downregulated changes in the expression at the mRNA and protein levels during CRC progression. Importantly, the analysis of mRNA expression of CaMK2δ in individual tissue samples was in agreement with the results of the large-scale analysis of the transcriptomic and proteomic datasets. Consistently, Feng et al. [28] determined the mRNA expression profile involved in the progression of *F. nucleatum*-induced CRC using the GeneChip Human Transcriptome Array 2.0. The Kyoto Encyclopedia of Genes and Genomes (KEGG) analysis and the protein–protein interaction network analysis of the major aberrantly expressed mRNAs indicated that both calcium signaling pathways and CaMK2β expression were downregulated in *F. nucleatum*-induced CRC specimens compared with paracancerous tissues. Furthermore, the genes differentially expressed between the colon and rectal cancer were detected and identified using oligonucleotide microarray analysis and the TwoClassDif method, respectively [29]. The results showed that CaMK2γ expression was significantly reduced in rectal cancer tissues. Collectively, these bioinformatic findings illustrate the essential involvement and potential role of CaMK2 in the development of CRC. Thus, much effort is needed to explore the precise functional effects and underlying regulation of CaMK2 in CRC tumorigenesis.

### The altered CaMK2 in glioblastoma multiforme (GBM)

In addition to CRC, similar results have been found in gliomagenesis. Johansson et al. [30] attempted to identify and validate differentially expressed genes in PDGF-induced glioma in mice using microarray analysis of RNA expression profiling and quantitative real-time PCR, respectively. Their results showed that CaMK2β was strongly downregulated in tumors compared to that in normal brain tissue. Consistent with this view, Xiong et al. [31] intersected the target genes of the differentially expressed miRNAs in GBM that were collected from the gene expression omnibus (GEO) database and the GBM-associated genes that were obtained from the gene expression profiling interactive analysis (GEPIA). Results from KEGG analysis of the overlapping genes suggested that both CaMK2β and CaMK2γ were centralized in the glioma pathway. Data from GEPIA showed that the expression of CaMK2β and CaMK2γ in GBM tissues was lower than that in the normal control.

Additionally, the possible involvement of CaMK2 in the recurrence and prognosis of GBM has also been reported. For example, researchers compared and corroborated the transcriptional profiles of eight pairs of primary low-grade gliomas and corresponding recurrent high-grade gliomas from patients using oligonucleotide-based microarray analysis and real-time RT-PCR analysis [32]. Their results confirmed that the transcript level of CaMK2γ in recurrent high-grade gliomas was significantly decreased compared with that in primary low-grade gliomas. More importantly, CaMK2γ transcript levels in high-grade glioblastomas were also significantly decreased compared to low-grade astrocytomas in another independent set of 43 gliomas. These results suggest that the reduced CaMK2γ may have a role in the recurrence of GBM, and the low CaMK2γ transcript level might be a poor prognostic indicator for gliomas. In contrast, other researchers screened and identified prognosis-associated differentially expressed genes in glioblastoma using microarray data of tumor and normal tissue samples downloaded from the TCGA dataset and GEO dataset GSE22866 [33]. Subsequently, they established a prognostic prediction system using Bayes discriminant analysis, which was successfully validated by the microarray data of patients with different prognoses from the TCGA dataset, a Chinese Glioma Genome Atlas (CGGA) dataset, and another GEO dataset. Finally, their results showed a close association between high CaMK2α mRNA expression and a poor prognostic factor for GBM.

Taken together, these studies have preliminarily indicated that the prominently altered CaMK2 transcript level may be closely associated with the tumorigenesis and recurrence of GBM and that CaMK2 is a potential candidate for predicting GBM prognosis.

### The correlation of CaMK2 with therapy sensitivity and prognosis in cancers

In terms of drug tolerance, some research has preliminarily shown a positive correlation between CaMK2 and drug resistance or prognosis of cancer using bioinformatics approaches. Lian et al. [34] identified the differentially expressed genes between TPF-responsive primary HSCC patients and resistant patients using mRNA microarray analysis and found that the expressions of CaMK2α and CaMK2β were upregulated in TPF-resistant primary HSCC patients compared to the sensitive cases. The qRT-PCR results revealed that the expression of CaMK2α was significantly increased in FaDu cells (a TPF-sensitive HPC cell line) after exposure to TPF treatment. This suggests that high CaMK2α expression was positively related to TPF tolerance in HSCC. Similarly, researchers applied a tandem mass tag (TMT)-based quantitative proteomics approach to distinguish the differentially expressed proteins between the chemotherapy-sensitive and chemotherapy-resistant plasma samples from metastatic TNBC patients and found that the protein levels of CaMK2α and CaMK2β in the resistance group were significantly increased compared to the sensitive group [35]. In agreement with the proteomic analysis, the higher level of CaMK2α in the resistance group was further validated by ELISA. Notably, metastatic TNBC patients with higher CaMK2α levels had shorter overall survival than those with lower CaMK2α levels. These analytical data indicate that CaMK2 (especially α isoforms) may serve as a potential biomarker for predicting poor chemotherapy outcomes and prognosis in metastatic TNBC. Consistent with this opinion, Tang et al. [36] investigated the prognostic implications of genetic variants in early-stage NSCLC patients by genome-wide analysis and found that the variant allele of rs10023113 in CaMK2δ was significantly associated with the poor prognosis of NSCLC. Altogether, these bioinformatic studies preliminarily raise the possibility that CaMK2 is involved in regulating therapy-tolerance of cancer. Thus, future in-depth studies are required to clarify the role of CaMK2 in drug resistance and cancer prognosis.

### The epigenetic modification of CaMK2 in cancer

In terms of epigenetics, the CaMK2 promoter has been reported to undergo methylated modification. Genes showing downregulation in breast cancer cells were screened and selected by a series of in silico analyses from the Gene Expression Atlas interface. Then, these in silico screened genes underwent real-time methylation-specific PCR (MSP) and RT-PCR [37]. The results showed that the promoter of CaMK2β was hypermethylated and that the expression of CaMK2β was downregulated in breast cancer cell lines. More importantly, induction of CaMK2β promoter demethylation using a methyltransferase inhibitor (5-Aza-2′-deoxycytidine) remarkably upregulated CaMK2β expression in breast cancer cells. These results indicated that the downregulation of CaMK2β expression was partially attributed to hypermethylation at the CaMK2β promoter in breast cancer cells. In support of this view, a study reported that significant methylation of the CaMK2 promoter was identified in lingual mucosa samples from a lingual SCC mouse model using microarray and methylated DNA immunoprecipitation sequencing (MeDIP-Seq) analysis. This suggests that CaMK2 expression might be decreased in lingual carcinogenesis [38]. Moreover, the CaMK2 fusion protein has also been reported to participate in carcinogenesis. Wang et al. [39] identified fusion proteins involved in esophageal adenocarcinoma (EAC) using next-generation RNA sequencing and PCR verification in the EAC specimens and adjacent nonmalignant specimens from 40 patients and found that the USP54-CaMK2γ fusion transcript was present in the EAC specimens but not in the adjacent nonmalignant tissues. This finding indicates that CaMK2γ may play a functional role in EAC carcinogenesis through the formation of the USP54-CaMK2γ fusion protein.

In summary, these bioinformatic analyses using different approaches have commonly demonstrated the significantly altered expression of CaMK2 in cancer progression. This indicates that CaMK2 might contribute to the pathogenesis of cancer. However, the conclusions reached in the above papers are not very consistent, and most studies were only performed using different bioinformatics approaches. Therefore, the precise and potentially functional effects of CaMK2 on cancer progression need to be further investigated in comprehensive experiments. Of note, the isoforms of CaMK2 in distinct cancers are different. Thus, we speculate that the prominent isoform of CaMK2 is dependent on the tumor type and that the different isoforms of CaMK2 may produce synergistic or antagonistic effects on cancer progression. Of course, our assumption needs to be determined through in-depth experiments in vivo and in vitro.

### The role of CaMK2 in the cellular proliferation, migration, and metastasis of cancer

Unsurprisingly, CaMK2 has been demonstrated to be implicated in the regulation of cell proliferation, migration, and metastasis in a variety of cancer types by a series of experimental studies (Table 2).

**Table 2:** The calcium/calmodulin-stimulated protein kinase II regulates the cellular proliferation, migration, invasion and metastasis in various cancer types

Cancer type	Cell lines	The functional influence of CaMK2 on cancer progression	References
CRC	HCT116 cells HT29 cells	CaMK2γ significantly inhibites cell cycle arrest, decreases apoptosis, thus promoting cell growth	[40]
	HCT116 cells	CaMK2 activity is required for cellular proliferation, migration and invasion	[41]
Breast cancer	11q13-amplified breast cancer cells	CaMK2 signaling is required for the ANO1-mediated cell survival and proliferation	[42]
	MDA-MB-231 cells MCF-7 cells	CaMK2α activation positively regulates cellular growth, migration and invasion	[43]
	MDA-MB-231 cells	The dephosphorylation of CaMK2α at T253 accelerates the metaphase–anaphase transition and increases cell proliferation	[44]
Gastric cancer	BGC-803 cells	CaMK2α activation promotes proliferation and metastasis	[45]
	BGC-823 cells	Inhibition of CaMK2β decreases cell viability, survival and migration	[46]
Hepatocarcinoma	Hep3B cells HepG2 cells	Inhibiting CaMK2 activity using KN-62 decreased the protein synthesis and functionally activity of HIF-1α in hepatocellular carcinoma cells	[47]
	Huh7, MHCC97H; SNU398; SK-Hep-1	Inhibiting CaMK2γ with KN93 and shRNAs significantly reduced survival and proliferation in carcinoma cells, wheras, overexpression of CaMK2γ exerts an opposite effect	[48]
Prostate cancer	C4-2B cells	Inhibition of CaMK2 activity results in significant inhibition of cell proliferation	[49]
	LNCaP cells	Overexpression of CaMK2 (α and β isofroms) decreases apoptosis and promotes cell growth	[50]
		KN-93 synergistically increases cell death in combination with low doses of doxorubicin and converts the phenotype of prostate cancer cells from TRAIL-resistant to TRAIL-sensitive	[52]
	C4-2B4 cells PC3-mm2 cells	CaMK2 activation promotes cell survival, growth, migration and metastasis in vivo and vitro	[51]
	PC3 cells	Inhibition of CaMK2 (α isoform) using AIP or a dominant negative mutant significantly mitigates H_2_O_2_-induced cell death	[53]
Osteosarcoma	MG-63 cells 143B cells	Inhibiting CaMK2α using KN93, specific siRNA or the K42M kinase-dead construct (CaMK2α K42M) significantly decreases growth of osteosarcoma cells through the induction of p21-dependent cell cycle arrest	[54]
	MG-63, 143B, HOS, MNNG/HOS cells	Knockdown of CaMK2α decreases proliferation, migration and invasion in vitro and tumor burden in vivo; wheras, overexpression of CaMK2α has the opposite effects	[55]
Myeloid leukemia	K562 cells	Inhibition of CaMK2 activity with pharmacologic agents, dominant-negative constructs or shRNAs reduces the proliferation of myeloid leukemia cells	[56]
		overexpression of CaMK2γ greatly reversed berbamine-induced growth inhibition of myeloid leukemia cells in vivo and vitro	[57]
T cell lymphoma	H9 cells, SU-DHL-1 cells, JB6 cells, and Jurkat cells	CaMK2γ promotes lymphomagenesis and cellular proliferation of T cell lymphoma by regulating c-Myc protein expression	[58]
OSCC	HSC-3 and SAS cells	CaMK2 signaling may be involved in OSCC progression	[59]
GBM	U87MG, U251 and LN229 cells	CaMK2α might exert an inhibitory effect on cell survival and progression of GBM	[60]

*CRC* colorectal cancer, *HIF-1* hypoxia inducible factor-1, *OSCC* oral squamous cell carcinoma, *GBM* glioblastoma multiforme

### CaMK2 in colon cancer

In recent years, evidence suggests that CaMK2 signaling plays a positive role in regulating cell growth and migration in colorectal cancer (CRC) cells. A previous study showed that the SOCE inhibitor SKF-96365 significantly triggered cell cycle arrest and induced apoptosis. Thus, inhibiting the growth of colorectal cancer cells, with concomitant inhibition of the CaMK2γ signaling cascade. Moreover, CaMK2γ overexpression markedly abolished the inhibitory effects of SKF-96365 on cancer cells. More importantly, the direct genetic or pharmacological inhibition of CaMK2γ using siRNA or KN93 resulted in similar observations in SKF-96365-treated cells. These indirect and direct results suggest that CaMK2γ signaling is a critical mediator in regulating the growth of CRC cells [40]. Consistently, in another study, researchers found that the expression of CaMK2 was significantly increased in colon cancer samples and highest in poorly differentiated colon cancer specimens compared to paracancerous tissues. Inhibiting CaMK2 activity using KN93 remarkably repressed proliferation and attenuated migration and invasion in colon cancer cells. This biological effect may be dependent on the inhibition of ERK1/2 and p38 pathways [41]. Altogether, these experimental studies imply that CaMK2 plays an important role in the promotion of cellular growth, migration, and invasion in CRC.

### CaMK2 in breast cancer

The biological role of CaMK2 in breast cancer progression has been preliminarily examined in several recent studies. First, a previously published paper showed that inhibition of CaMK2 signaling partially abrogated ANO1 promotion of cell viability and proliferation in ANO1-amplified and -overexpressed breast cancer, suggesting that CaMK2 plays a critical role in cellular proliferation and oncogenesis in breast cancer [42]. In support of this view, a later study found that the expression and phosphorylation at T286 of CaMK2 in breast cancer specimens and its lymph node metastasis tissues were significantly increased. Pharmacological inhibition of CaMK2 using both AIP and KN-93 decreased the migration and invasion of highly aggressive MDA-MB-231 cells. In contrast, overexpression of the constitutively active (T286D phosphomimetic mutant) form of CaMK2α significantly increased the growth, migration, and invasion capacities of MDA-MB-231 and MCF-7 breast cancer cells. Induction of the T286D phosphorylation of CaMK2α effectively promoted epithelial-mesenchymal transition (EMT) and up-regulated the phosphorylation of FAK, STAT5a, and Akt. Therefore, data from this study suggest that the stimulatory effects of CaMK2α-T286D on breast cancer cells are closely associated with enhanced EMT and increased activation of FAK, STAT5a, and Akt [43].

Notably, CaMK2 has also been demonstrated to undergo functional phosphorylation at other sites to regulate the proliferation and cell cycle of cancer cells [44]. In contrast to the phosphorylation of CaMK2 at T286, endogenous CaMK2 was dephosphorylated at T253 during the G2 and/or M phases of the cell cycle. Overexpression of a T253V phosphonull form of CaMK2α significantly increased the proliferation rate of MDA-MB-231 cells. In contrast, overexpression of the phosphomimetic T253D, but not the T286D, significantly reduced proliferation, inhibited metaphase-anaphase transition, and induced cell apoptosis. This result indicated that the dephosphorylation of CaMK2α at T253 was essential for controlling the cell cycle, specifically the metaphase–anaphase transition.

Taken together, these reports show that CaMK2, especially the α isoform, is critical for growth, migration, and invasion in breast cancer, and that phosphorylation of CaMK2α at different sites has a diverse influence on the cell cycle and aggressive phenotypes of breast cancer cells.

### CaMK2 in gastric cancer

Recently, the function and underlying mechanism of CaMK2 in the control of gastric cancer cell growth and metastasis have been reported. A previous study found that high CaMK2 phosphorylation at Thr286 was expressed in gastric cancer tissues with metastasis and in four higher metastatic gastric cancer cell lines. This suggests that CaMK2 activation may be involved in the regulation of gastric cancer cell metastasis. As expected, overexpression of CaMK2α H282R (a constitutively active CaMK2α) significantly accelerated cell proliferation, migration, and invasion, with a concomitant increase in NF-κB and Akt-mediated matrix metalloproteinase-9 (MMP-9) production. However, inhibition of CaMK2 activity using KN‑62 exerted the opposite effects. These experimental findings indicate that CaMK2-mediated promotion of migration and invasiveness in gastric cancer cells might rely on NF-κB and Akt-dependent MMP9 production [45]. Consistently, a later study also demonstrated that blockade of CaMK2β using a specific inhibitor (KN93) or shRNAs remarkably inhibited proliferation and migration of gastric adenocarcinoma cells. This inhibitory effect might be related to the decreased phosphorylation of NF-κB, AKT, S6, and mTOR [46]. Collectively, these results represent a positive function and possible mechanisms of CaMK2 in the regulation of gastric cancer growth and metastasis. Thus, offering a potential target for the prevention of gastric cancer metastasis.

### CaMK2 in liver cancer

Similarly, the essential role of CaMK2 in HCC progression has recently been reported. First, the potential effect of CaMK2 on liver cancer was indirectly assessed by a simple experiment [47]. KN-62, a pharmacological inhibitor of CaMK2, specifically and effectively suppressed protein synthesis and the functional activity of hypoxia-inducible factor (HIF)-1α in hepatoma cells during hypoxia. Given that HIF-1α is a transcription factor that contributes to the angiogenesis and growth of tumors in the hypoxic microenvironment, it is reasonable to speculate that CaMK2 can promote the growth and survival of hepatocellular carcinoma cells. In support of this possibility, Meng et al. [48] proved that phosphorylated (activated) CaMK2γ was frequently present in liver tumor samples compared with the adjacent peritumor tissues. The frequency of CaMK2γ phosphorylation in liver cancer specimens was positively correlated with the clinical stages of hepatocarcinoma. This suggests that CaMK2γ plays a critical role in the development of hepatocarcinoma. As expected, inhibition of CaMK2γ using KN93 or shRNAs considerably decreased the survival and growth of hepatoma cells in vivo and in vitro. Whereas, overexpression of CaMK2γ promoted cell proliferation in vitro. Importantly, berbamine could recapitulate this inhibitory phenotype by directly targeting CaMK2γ. Results from this experiment showed that CaMK2 (mainly the γ isoform) plays a significant role in the growth and development of liver cancer.

Therefore, these indirect and direct consequences collectively indicate that CaMK2, especially the γ isoform, could facilitate the survival, proliferation, and growth of liver cancer cells.

### CaMK2 in prostate cancer

Accumulated evidence has emphasized the essential role of CaMK2 in regulating the growth, invasion, and metastasis of prostate cancer cells. A previous study reported that inhibition of CaMK2 activity using a pharmacological inhibitor (KN93) significantly repressed cell proliferation and invasion of C4-2B cells. This effect was highly associated with inhibition of Notch-1 signaling [49]. Consistent with this view, another study found that inhibition of CaMK2 activity, KN-93, induced dose-dependent apoptosis in LNCaP cells. Overexpression of CaMK2 (α and β isoforms) sharply diminished apoptosis and increased cell growth of LNCaP under steroid‑free conditions [50]. Subsequently, an original experiment published in *Cancer Research* directly comprehensively demonstrated the important role of CaMK2 in enhancing prostate cancer progression. Active CaMK2 was highly expressed in metastatic prostate cancer specimens and more tumorigenic PC3-mm2 cells. Genetic deletion of CaMK2 (all four CaMK2 isoforms) in PC3-mm2 cells using the CRISPR/Cas9 system reduced cell survival under low serum conditions, anchorage-independent growth and migration, and lymph node metastasis. However, these altered biological phenotypes were significantly rescued by re-expression of CaMK2-T286D (a constitutively active form of CaMK2) in CaMK2 knockout PC3-mm2 cells. Correspondingly, overexpression of CaMK2-T286D in the less tumorigenic C4-2B4 cells significantly promoted these phenotypes. In addition, β-oxidation-generated acetyl-CoA could enhance prostate cancer cell survival, migration, and metastasis by binding to the CaMK2 regulatory domain and increasing CaMK2 activity [51]. Furthermore, researchers have also shown that KN-93 could increase apoptosis and induce cell death when other agents fail to kill prostate cancer cells after androgen deprivation. More importantly, the combination of KN-93 and low doses of doxorubicin further induced cell death and also converted the phenotype of prostate cancer cells from TRAIL-resistant to -sensitive. This indicates that CaMK2 may be a promising target for drug tolerance therapy in prostate cancer [52]. Together, the results from these reports collectively indicate that the synthetic drugs and endogenous genes that decrease CaMK2 production or inactivate CaMK2 may have the potential to inhibit prostate cancer progression.

Contrary to the mainstream opinion, one previous study elucidated the negative role of CaMK2 in cancer cell survival [53]: pharmacological inhibition or dominant-negative mutant of CaMK2 remarkably alleviated H_2_O_2_-induced cell death in PC3 cell. This suggests that CaMK2 has the ability to promote cell death under oxidative stress.

### CaMK2 in osteosarcoma

Osteosarcoma is the most frequent type of primary bone cancer in humans and lacks specific molecular targets for potential therapeutic options. In recent years, the α isoform of CaMK2 has been shown to positively regulate the aggressive phenotypes of human osteosarcoma. First, a published study explored the critical role of CaMK2α in the growth of osteosarcoma [54]. Primary osteosarcoma tissues and human osteosarcoma cell lines expressed high levels of total and phosphorylated forms of CaMK2α. This suggests that the expression and activity of CaMK2α are increased in human osteosarcoma. Inhibition of CaMK2α using a pharmacological antagonist (KN93), specific siRNA, or the K42M kinase-dead construct (CaMK2α K42M) resulted in a significant decrease in the growth of osteosarcoma cells. This inhibitory phenotype was due to the induction of p21-dependent cell cycle arrest. Similar to the in vitro results, KN-93 treatment of mice xenografted with human osteosarcoma cells dramatically decreased tumor volume and size. This indicates that inhibition of CaMK2 activity also arrested the in vivo growth of human osteosarcoma. Soon afterward, the promotion of CaMK2α on the metastasis and tumorigenesis of human osteosarcoma was further confirmed by another experiment [55]. Knockdown of CaMK2α by lentivirus shRNA in aggressive osteosarcoma MG-63 and 143 B cells significantly decreased proliferation, migration, and invasion in vitro and reduced tumor burden in vivo; in contrast, overexpression of CaMK2α by retrovirus in nonaggressive and nontumor forming osteosarcoma HOS cells dramatically increased cell proliferation, migration, and invasion in vitro, resulting in tumor formation in vivo. Taken together, these experiments suggest that CaMK2α plays a critical role in determining the tumorigenic properties of osteosarcoma, and its inhibition might be a promising therapeutic target to combat this devastating disease.

### CaMK2 in leukemia

In addition to solid tumors, CaMK2 has also been demonstrated to positively regulate cancer progression in several types of leukemia. In the field of chronic myeloid leukemia (CML), a previous study published in *Cancer Research* showed that autophosphorylated (activated) CaMK2γ was predominantly and commonly present in primary acute myelogenous leukemia samples and different myeloid leukemia cell lines. Inhibition of CaMK2γ with pharmacologic inhibitors, dominant-negative constructs, or shRNAs resulted in a significantly reduced proliferation of myeloid leukemia cells. This effect was accompanied by inactivation/downregulation of the MAPK, JAK/Stat, Stat3/Stat5, and GSK3β/β-catenin pathways. Thus, the effect of CaMK2γ on proliferation in myeloid leukemia cells is likely mediated through the regulation of multiple critical signaling pathways [56]. In agreement with these findings, overexpression of CaMK2γ not only significantly attenuated berbamine-induced inhibition of leukemia cell proliferation in vitro, but also markedly reversed berbamine-induced growth inhibition of xenograft tumors in vivo [57]. Together, CaMK2γ contributes to cellular proliferation in myeloid leukemia.

Additionally, another study published in *Cancer Cell* regarding T-cell lymphoma (TCL) showed that CaMK2γ genetic deletion significantly inhibited the development of *N*-methyl-*N*-nitrosourea (MNU)-induced TCL in mice. It also delayed the tumorigenic kinetics of the bone marrow transplantation (BMT) experiment of hematopoietic progenitors infected with retroviruses driving the expression of an oncogenic constitutively active form of Notch1 (Notch1-△E). This suggests that CaMK2γ is required for T cell lymphomagenesis in vivo. Consistently, both inhibition of CaMK2γ activity by KN93 and genetic ablation of CaMK2γ by CRISPR/Cas9 technology dramatically induced G2/M arrest, increased apoptosis, and impaired cellular proliferation. This was consistent with a reduced protein level of c-Myc. In contrast, CaMK2γ overexpression significantly promoted proliferation and colony formation. Notably, c-Myc overexpression reversed the effect of CaMK2γ deletion on decreased cellular proliferation [58]. Taken together, these results indicate that CaMK2γ positively regulates tumorigenesis and cell proliferation of TCL by sustaining c-Myc levels.

Altogether, these previous experiments suggest that CaMK2γ regulates cellular proliferation and tumorigenesis in leukemia and represents a novel potential target for leukemia therapy.

### CaMK2 in other cancers

Finally, the possible involvement of regulation of CaMK2 signaling in the progression of oral squamous cell carcinoma (OSCC) and glioblastoma multiforme (GBM) has also been reported in two other studies. One of these studies showed a positive correlation between CaMK2 activation and cancer progression in OSCC [59]. Synaptotagmin12 (SYT12) expression in primary OSCC tissue and OSCC-derived cell lines was significantly increased compared to that in normal tissues. SYT12 knockdown in OSCC cells effectively inhibited cellular proliferation, migration, and invasion, which was accompanied by a remarkable reduction in CaMK2-phosphorylation. This finding suggests that CaMK2 signaling may be involved in the malignant phenotype of OSCC. Therefore, the direct functional effect and underlying mechanism of CaMK2 on OSCC development need to be investigated in a comprehensive experiment.

In contrast, the experimental results from another study showed a negative correlation between CaMK2 activation and cell viability in GBM [60]. The phosphorylated CaMK2α at T286 in primary GBM tissues and its tumor cell lines were significantly reduced compared to their normal counterparts. Moreover, the level of phospho CaMK2α-T286 in high-grade GBM (grade IV) was much lower than that in the lower grades. This suggests that inhibition of CaMK2α phosphorylation was required for GBM progression to an aggressive and malignant phenotype. Importantly, the bacoside treatment caused non-apoptotic cell death in GBM cell lines with a concomitant significant increase in phospho-CaMK2α. These experimental data indicate that activated CaMK2α may exert an inhibitory effect on the survival and progression of GBM. Paradoxically, the bioinformatics data from this study showed that the high transcriptional level of CaMK2α was closely associated with a poor prognosis of GBM. This suggests that CaMK2α might have the ability to promote the progression and malignancy of GBM. Thus, the precise and direct functional role of CaMK2α in GBM progression is not fully understood.

In summary, these observations collectively highlight the critical role of CaMK2 in promoting cellular survival, proliferation, migration, and metastasis in a series of cancer types. This indicates that CaMK2 is a key oncogenic factor contributing to cancer progression. CaMK2 offers a promising target for novel potential therapies to prevent cancer progression. Compounds that inhibit CaMK2 signaling might be attractive therapeutic agents for the treatment of malignant diseases. It is noteworthy that the different phosphorylations of CaMK2 have diverse influences on cancer progression. Therefore, understanding the comprehensive molecular mechanism of CaMK2 in cancer may help to improve the efficacy of these agents.

### The influence of CaMK2 on cancer stem-like traits

Cancer stem-like cells (CSCs) or cancer-initiating cells (TICs) are a subpopulation of cells with high tumorigenic potential, self-renewal ability, and prominent expression of stemness-specific markers such as CD133, Nanog, Sox2, and Oct4. Studies have implied that CSCs are responsible for cancer initiation, malignant progression, therapeutic resistance, and relapse [61–63]. Thus, specifically targeting CSCs may be a promising approach to cure refractory cancer [64]. Recently, some studies have identified the functional effect of CaMK2 on CSCs in several different types of cancer (Table 3).

**Table 3:** The calcium/calmodulin-stimulated protein kinase II regulates cancer stem-like features in several cancer types

Cancer	Cell lines	Method of selecting CSCs	The functional influence of CaMK2 on CSCs/TICs	References
Liver cancer	Huh7, MHCC97H, SNU398	CD133^+^ Huh7 population and CD90^+^ MHCC97H population were sorted by FACS	CaMK2 inhibitor, KN93, remarkably decreased the stem cell populations and inhibited sphere formation in liver cancer cell lines	[48]
GBM	U87MG U373MG	GSCs were enriched by neurosphere formation	Inhibiting CaMK2γ remarkably repressed the stem cell-like phenotypes as well as the expression of stemness markers by decreasing c-Met signaling pathway	[66]
	Patient-derived xenografts	BTICs and non-BTICs were isolated based on functional criteria	CaMK2 was highly expressed in non-BTICs and positively associated with the non-BTICs phenotypes	[67]
CML	K562 KCL22	CD34^+^/CD38^−^ leukemia stem cells were sorted from primary CML samples using FACS	CaMK2γ was highly up-regulated and activated in leukemia stem cells of CML	[57]
Breast cancer	MCF-7, MDA-MB-231	long-term glucose deprivation	CaMK2α inhibition using siRNAs or KN62 significantly increased apoptosis and decreased survival of CSCs	[68]
Lung cancer	HCC827, A549, H1299 ZRLC-1	CSCs were enriched by oncosphere culture	CaMK2γ significantly enhanced the stem-like traits and tumorigenicity of lung cancer cells in an Akt- and β-catenin-dependent manner	[69]

*CSCs* cancer stem cells, *GBM* glioblastoma multiforme, *GSCs* glioblastoma stem-like cells, *BTICs* brain tumor initial cells, *CML* chronic myeloid leukemia, *FACS* fluorescence-activated cell sorting

### The effect of CaMK2 on stemness in liver cancer

Interestingly, two papers from the same laboratory team showed a contradictory effect of CaMK2 on stem-like traits in liver cancer. Results from the in vitro assay showed that inhibition of CaMK2 activity using the chemical inhibitor KN93 in CD133 + liver cancer cells sufficiently induced cell death. KN93 significantly reduced the percentage of CD133 + /CD90 + stem cell populations and strongly inhibited hepatosphere formation in liver cancer cell lines. Importantly, berbamine and its derivative bbd24 mimicked the effects of KN93 on liver cancer-initiating cells by targeting CaMK2 signaling [48]. Together, data from in vitro experiments suggested that CaMK2 plays a significant role in maintaining the stemness and self-renewal ability of liver cancer stem cells. In contrast to the oncogenic role of CaMK2 in liver cancer cells in in vitro experiments, the genetic deletion of CaMK2γ in mice resulted in significantly enhanced chemical-induced hepatocarcinogenesis unexpectedly [65]. Mechanistically, CaMK2γ deletion could promote several key causes of liver tumor initiation, including severe cell death and inflammation, the continuous compensatory proliferation of premalignant hepatocytes, and activation of the AKT/mTORC1 pathway. Therefore, results from in vivo experiments surprisingly indicated that CaMK2γ inhibited tumorigenesis in a model of DEN-induced hepatocellular carcinoma, and also played an inhibitory role in the stemness of liver cancer.

The reasons for this contradictory conclusion should be fully addressed. Considering that KN93 has the ability to inhibit CaMK2 activity, including α, β, γ, and δ isoforms. We speculate that the absolute genetic deletion of CaMK2γ compensatorily increases the expression of other CaMK2 isoforms. This leads to enhanced stem-like phenotypes in liver cancer. Of course, our speculation needs to be fully investigated through an in-depth experiment.

### The effect of CaMK2 on stemness in GBM

Similarly, two published papers have also indicated an inconsistent relationship between CaMK2 and stem-like features in GBM. A recent study found that CaMK2γ inhibition significantly suppressed not only the stem-like traits of GBM cells, such as cell growth and neurosphere formation, but also the protein levels of GSC stemness markers, such as CD133, Nanog, Sox2, and Oct4 [66]. Notably, HBC, a synthetic curcumin derivative, hydrazinobenzoyl-curcumin, recapitulated the suppressive effects of CaMK2γ inhibition on GBM stemness by blocking the CaMK2-dependent c-Met signaling pathway. Thus, these findings indicate that CaMK2γ plays a critical role in sustaining the stem-like features of GBM cells, and targeting CaMK2γ might be a novel promising approach for GSC therapy. In contrast to this view, another paper published in *Nature Neuroscience* showed a negative relationship between CaMK2 and stemness in GBM [67]. First, CaMK2 was highly expressed in non-BTICs (brain tumor-initiating cells). Second, CaMK2 might sustain the non-BTICs phenotypes by inhibiting Drp1 activity. Last, the higher expression of CaMK2 (α or γ isoform) was associated with longer patient survival.

Although the correlation of CaMK2 and stemness in GBM is not consistent, these currently direct and indirect results have, at least, collectively confirmed the involvement of CaMK2 in regulating the stemness of GBM. The accurate role of CaMK2 in the stem-like traits of GBM needs to be further elucidated.

### The effect of CaMK2 on stemness in other cancers

In addition, the main positive correlation between CaMK2 and stemness in several cancers has been preliminarily explored. In chronic myeloid leukemia (CML), both total and phosphorylation levels of CaMK2γ were highly increased in CD34 + /CD38− leukemia stem cells (LSCs) but not in CD34- CML cells and normal hematopoietic cells (HSCs). This was concomitant with the activation of multiple LSC-related signaling pathways including NF-kB, Wnt/β-catenin, and Stat3 pathways. These results suggest that CaMK2γ might play a positive role in the survival of CML cancer stem cells [57]. Therefore, it is worthwhile to directly investigate the functional effect of CaMK2γ on the stem-like features of CML using a comprehensive in-depth experiment in the future. Consistently, a study on breast cancer showed that metabolic stress-resistant CSCs exhibited significantly increased antiapoptotic capability and expressed high levels of phosphorylated CaMK2α during prolonged glucose deprivation compared with their parental lineages. Inhibiting CaMK2α with specific siRNAs or pharmacological inhibitor (KN62) remarkably increased apoptosis and impaired survival of CSCs in response to prolonged glucose deprivation, which was accompanied by a downregulation of NF-κB-dependent SERCA2 expression. Together, these data indicate that CaMK2α is crucial for regulating survival in metabolic stress-resistant CSCs by increasing NF-κB-dependent SERCA2 expression in breast cancer [68]. Similarly, an experiment on lung cancer demonstrated that CaMK2γ was aberrantly activated and expressed in highly tumorigenic stem-like lung cancer cells, and was also closely correlated with poor prognosis in human lung cancer. Functionally, CaMK2γ promoted stem-like properties of lung cancer cells, including iPSC factor expression and oncosphere formation, in an Akt- and β-catenin-dependent manner. Moreover, CaMK2γ also enhanced the tumorigenic potential of lung cancer cells in an in vivo assay [69]. Therefore, these findings highlight the critical role of CaMK2γ in maintaining the stemness and tumorigenicity of lung cancer cells.

In summary, these previous studies have highlighted the critical role of CaMK2 in regulating cancer stemness in a series of cancer types. This suggests that targeting CaMK2 may be a promising approach to control stem-like features of cancer. Notably, the role of CaMK2 in regulating cancer stem-like traits in certain cancers is controversial. Thus, it is important for researchers to further explore the precise and specific functions and underlying mechanisms of CaMK2 in multiple cancer types.

### The influence of CaMK2 on drug resistance in cancer

In addition to its role of CaMK2 in cancer stemness, the potential effect of CaMK2 on therapeutic resistance has also been investigated. A previous preliminary study showed that artemisinin induced a doxorubicin-resistant phenotype in human colon carcinoma cells. However, inhibition of CaMK2 activity blocked the effect of artemisinin on doxorubicin accumulation and cytotoxicity. This suggests that CaMK2 activity is essential for artemisinin-induced resistance to doxorubicin in human colon cancer cells [70]. Consistent with the stimulatory effect of CaMK2 on drug resistance in human colon cancer, an experiment on hypopharyngeal carcinoma (HPC) reported that the mRNA level of CaMK2α was increased when FaDu cells were treated with TPF. This indicates that CaMK2α might be involved in TPF resistance. As expected, knockdown of CaMK2α using siRNA in FaDu cells led to a significantly decreased TPF IC50 [71]. These combined results suggest that CaMK2α also plays an important role in the TPF resistance of HPC. Furthermore, CaMK2δ overexpression decreased apoptosis and increased cell viability in human epithelial ovarian cancer cells under cisplatin treatment. This suggests that CaMK2δ contributes to cisplatin resistance in ovarian cancer cells [72]. Moreover, overexpression of CaMK2γ was directly proven to enhance the chemoresistance of liver cancer cells to 50-FU [48].

Although studies concerning the correlation between CaMK2 and therapeutic resistance in cancers are limited and plain, the current results have commonly elucidated the important role of CaMK2 in regulating drug resistance. Given that cancer stemness is the reason for therapeutic resistance and relapse, results from these drug-resistance assays have indirectly indicated the mainly positive role of CaMK2 in cancer stemness. Therefore, it is worthwhile to further explore the functional effect of CaMK2 on stemness in certain cancers through a comprehensive in-depth experiment.

## Conclusion

In the past decades, some purely bioinformatic evidence has shown the downregulated expression of CaMK2 in a variety of malignant tumors. This indicates that CaMK2 may be involved in cancer progression. Bioinformatics predominantly focuses on the transcriptional level changes of candidate genes in cancer. However, the changes in protein levels of candidate genes are more appropriate and significant for their functional roles in cancer progression. Therefore, it is important and necessary for researchers to accurately confirm the functional effect and underlying mechanism of candidate targets in cancer progression using a comprehensive in-depth experiment. Unexpectedly, results from experimental studies show that the protein levels of CaMK2 in several different cancer types are universally increased and that CaMK2 plays a vital role in promoting cancer progression, including growth, proliferation, invasion, and metastasis. The reasons for this inconsistent conclusion between bioinformatics and experiments are complex. In our opinion, the limited sample size and poor repeatability of published databases or microarray data in the bioinformatic analyses may partially explain this contradictory conclusion. Of course, the controversial findings might also be caused by the complicated and uncertain biological events between transcriptomics and proteomics. Therefore, bioinformatics analysis could be used as an effective tool to screen possible candidate genes involved in cancer development. However, the precise functional role and regulation of candidate proteins in cancer must be investigated by repeated in-depth experiments.

Recently, an association between CaMK2 signaling and cancer progression has emerged. A previous review described the structure and activation of CaMK2 and emphasized the role of CaMK2 in the regulation of cancer progression, especially proliferation, cell cycle, and metastasis [73]. Comparatively, we retrieved and replenished the latest experimental studies regarding the correlation of CaMK2 and cancer progression in the present review and found that CaMK2 plays a significant role in regulating the development of several cancer types, such as growth, proliferation, migration, invasion, and metastasis. Additionally, we have added some recently published articles focusing on the relationship between CaMK2 and cancer stemness or drug tolerance. Although the role of CaMK2 in cancer stemness is not completely consistent, most studies have shown that CaMK2 promotes cancer stem-like traits and confers drug resistance in cancer. These experimental results imply that CaMK2 may provide a novel opportunity for cancer treatment.

**Fig. 1:**
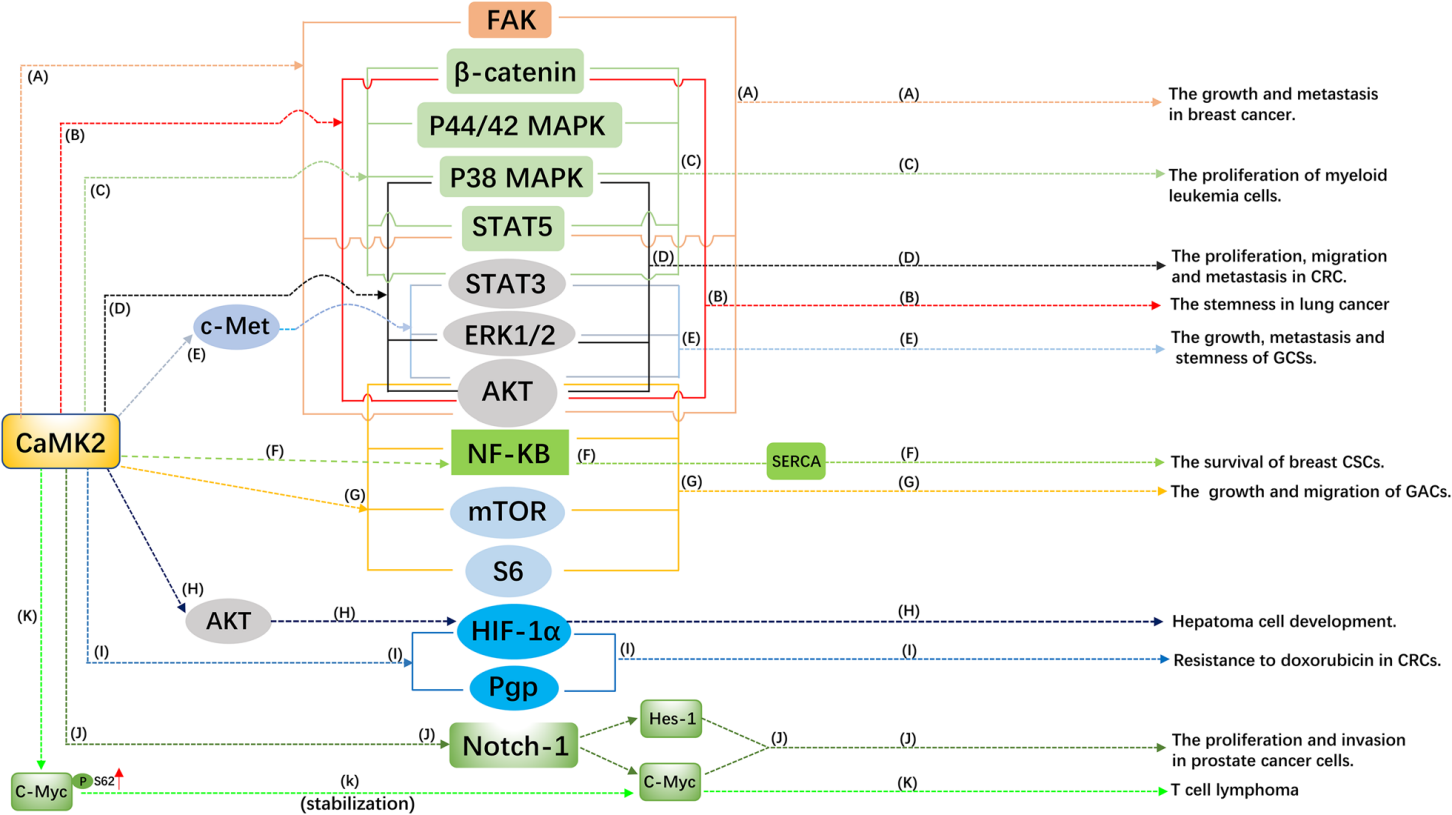
CaMK2 regulates the growth, metastasis, stemness and drug resistance of cancer by targeting multiple downstream substrates. A The activation of FAK, STAT5 and AKT are responsible for the stimulative effects of CAMK2 on celluar grwoth, migration and invasion in breast cancer [43]. B CaMK2γ accelerates the stem-like properties of lung cancer cells in an Akt- and β-catenin-dependent manner [69]. C CaMK2γ promotes myeloid leukemia cells proliferation through activation of p44/42 MAPK, p38 MAPK, STAT3, STAT5 and β-catenin [56]. D CaMK2γ facilitates the proliferation, migration and metastasis of colon cancer by activating several pathways including AKT, p38 MAPK and ERK1/2 [40]. E CaMK2γ enhances the growth, metastasis and stem-like traits of GSCs through upregulation of AKT, STAT3 and ERK1/2 pathways by activating c-Met signaling [66]. F CaMK2α maintains the breast CSCs survival in glucose-deprived conditions through SERCA induction by activating NF-kB [68]. G The activation of AKT, NF-kB, mTOR and S6 are required for the CaMK2β-mediated growth and migration of gastric adenocarcinoma cells [46]. H CaMK2 may promote hepatoma Cells development by AKT-dependent upregulation of HIF-1α [47]. I CaMK2 may induce resistance to doxorubicin in human colon cancer cells via activation of HIF-1α and Pgp [70]. J The functions of CaMK2 in promoting the celluar proliferation and invasion of prostate cancer are associated with the upregulation of Notch-1 signaling and its downstream substrates containing Hes-1 and c-Myc [49]. K CaMK2γ promotes T cell lymphoma by directly phosphorylating and stabilizing c-Myc protein [58]. The software used to generate the Figure is Microsoft Office PowerPoint (Office 2019 version). The signal network presented in this Figure is summarized from these experimental articles that included in this narrative review. *CRCs* colorectal cancer, *GSCs* glioblastoma stem-like cells, *GACs* gastric adenocarcinoma cells, *CSCs* cancer stem cells

Notably, the complex downstream signaling network of CaMK2 in regulating cancer progression and stemness is summarized and presented in Fig. 1. However, the upstream regulation of CaMK2 in cancer development remains unknown. Thus, more effort is needed to further elucidate the influence and underlying mechanisms of CaMK2 on cancer.

To conclude, CaMK2 is involved in cancer development and plays a significant role in the proliferation, metastasis, resistance, recurrence, and stemness in a range of cancer types. CaMK2 may be a promising target for cancer therapy.

## Data Availability

Not applicable.

## Abbreviations

PCR: Polymerase chain reaction
CRC: Colorectal cancer
GBM: Glioblastoma multiforme
GEPIA: Gene expression profiling interactive analysis
GEO: Gene expression omnibus
DEGs: Differentially expressed genes
DEMs: Differently expressed miRNAs
Lingual SCC: Lingual squamous cell carcinoma
MeDIP-Seq: Methylated DNA immunoprecipitation sequencing
EAC: Esophageal adenocarcinoma
USP54: Ubiquitin-Specific peptidase 54
HSCC: Hypopharyngeal squamous cell carcinoma
TPF: Docetaxel, cisplatin and 5-fluorouracil
TNBC: Triple negative breast cancer
TMT: Tandem mass tag
ELISA: Enzyme-linked immunosorbent assay
NSCLC: Non-small cell lung cancer
